# Crystal structure, Hirshfeld surface and energy framework analysis of bis­{3-(benzo­furan-6-yl)-5-[6-(1*H*-pyrazol-1-yl)pyridin-2-yl]-1*H*-1,2,4-triazol-1-ido}iron(II) methanol disolvate

**DOI:** 10.1107/S2056989025008655

**Published:** 2025-10-07

**Authors:** Illia Terpeliuk, Kateryna Znovjyak, Sergiu Shova, Olexandr V. Oksiuta, Vladimir M. Amirkhanov, Igor O. Fritsky, Maksym Seredyuk

**Affiliations:** a Department of Chemistry, Taras Shevchenko National University of Kyiv, Volodymyrska Street 64, Kyiv, 01601, Ukraine; b Department of Inorganic Polymers "Petru Poni" Institute of Macromolecular Chemistry Romanian Academy of Science Aleea Grigore Ghica Voda 41-A Iasi 700487 Romania; c Institute of Organic Chemistry, National Academy of Sciences of Ukraine, 5 Academik Kukhar Street, 02094, Kyiv, Ukraine; Venezuelan Institute of Scientific Research, Venezuela

**Keywords:** crystal structure, iron(II) complexes, neutral complexes, tridentate ligands

## Abstract

The title compound, [Fe(C_18_H_11_N_6_O)_2_]·2CH_3_OH, crystallizes in the ortho­rhom­bic space group *Pbcn* (No. 60) with half of the complex mol­ecule and a methanol mol­ecule in the asymmetric unit. In the complex, the two tridentate 3-(benzo­furan-6-yl)-5-[6-(1*H*-pyrazol-1-yl)pyridin-2-yl]-4*H*-1,2,4-triazol ligands meridionally bind to the central Fe^II^ ion through the N atoms of the heterocyclic groups, forming a pseudo-octa­hedral coordination sphere. In the crystal, C—H(pz)⋯π(ph) and C—H⋯N/C/O interactions consolidate the structure. Energy framework analysis at the B3LYP/6–31 G(d,p) theory level was performed to qu­antify the inter­action energies in the crystal.

## Abstract 2

The title compound, [Fe(C_18_H_11_N_6_O)_2_]·2CH_3_OH, crystallizes in the ortho­rhom­bic space group, with a distorted pseudo­octa­hedral Fe^II^ coordination sphere formed by two deprotonated tridentate 3-(benzo­furan-6-yl)-5-[6-(1*H*-pyrazol-1-yl)pyridin-2-yl]-4*H*-1,2,4-triazol ligands. In the crystal, mol­ecules stack in chains along the *b*-axis direction connected by weak C—H(pz)⋯π(ph) inter­actions and linked into layers by C—H⋯N/C/O inter­actions, which were qu­anti­fied by Hirshfeld surface and energy framework analysis.

## 1. Chemical context

3*d*-Metal complexes featuring tridentate bis­azole­pyridine ligands constitute a versatile class of coordination compounds with applications in biochemistry (Fares *et al.* 2020 ▸), catalysis (Wei *et al.*, 2015 ▸) and mol­ecular magnetism (Halcrow 2024 ▸). For ligands with asymmetric architectures, where one azole moiety bears a protonated nitro­gen heteroatom, deprotonation can balance the charge of the central metal ion, yielding neutral complexes. In this case, the peripheral substituents on the neutral complexes influence inter­molecular inter­actions, which in turn affect the connectivity, binding energy, crystal packing, and the coordination environment of the central ion.
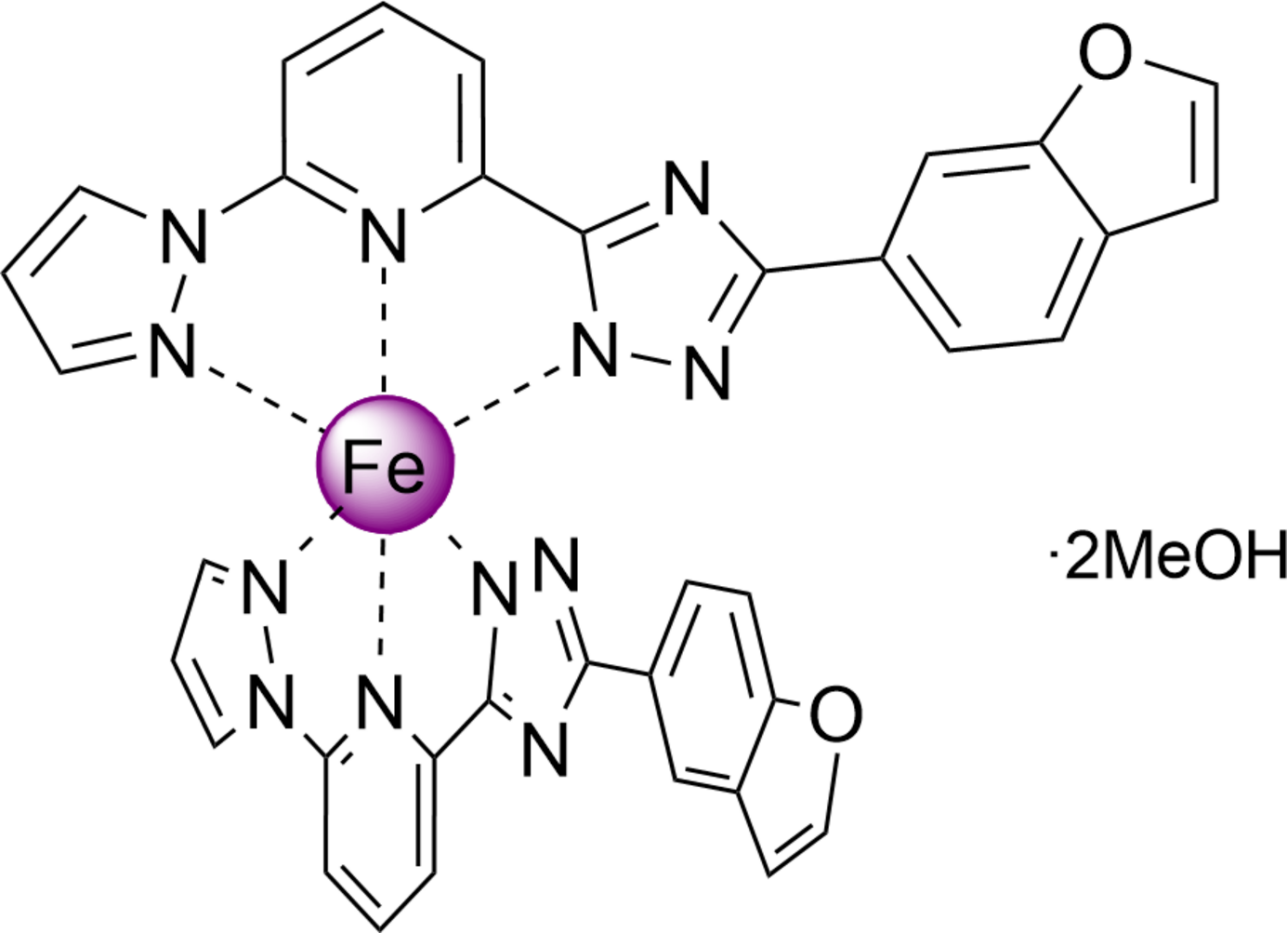


Given the prominence of bis­azole­pyridines as ligands in the Fe^II^ spin-crossover domain, and motivated by our longstanding inter­est in complexes of 3*d*-metals with N-heterocyclic ligands (Seredyuk *et al.*, 2007 ▸; Bonhommeau *et al.*, 2012 ▸; Piñeiro-López *et al.*, 2018 ▸), herein we report a new neutral low-spin complex based on the asymmetric ligand 3-(benzo­furan-6-yl)-5-[6-(1*H*-pyrazol-1-yl)pyridin-2-yl]-4*H*-1,2,4-triazol. This study details the synthesis and crystal structure of the title compound, incorporating benzo­furan groups to tune inter­molecular inter­actions.

## 2. Structural commentary

**Figure 1:**
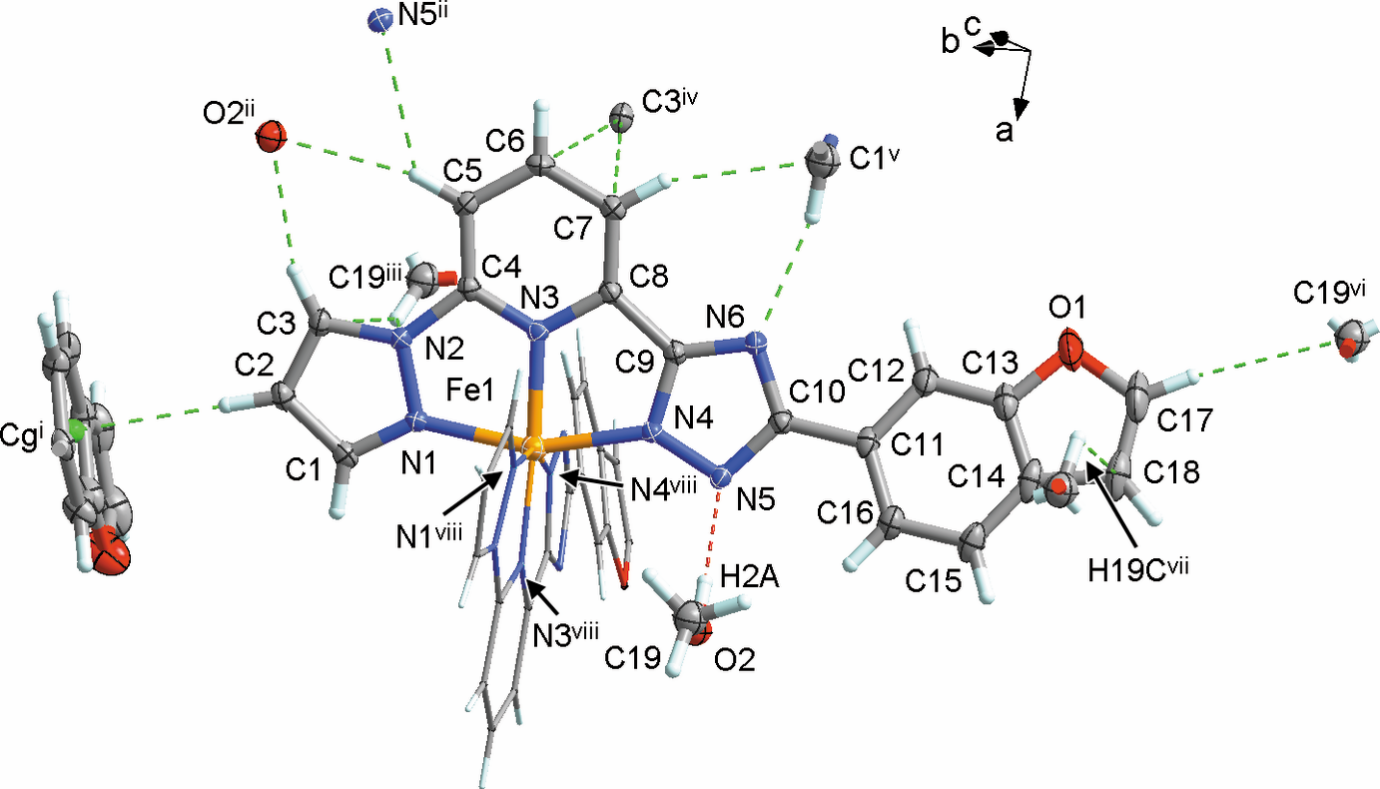
The mol­ecular structure in the asymmetric unit of the title compound and contact atoms with displacement ellipsoids drawn at the 40% probability level. The second ligand is shown in wireframe style for clarity. The strong O—H⋯N (red) and weak C—H⋯N/C/O/π (green) hydrogen bonds are shown with the nearest neighbours. Symmetry codes: (i) 1 − *x*, 1 + *y*, 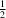 − *z*; (ii) 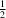 + *x*, 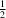 + *y*, 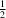 − *z*; (iii) 1 − *x*, *y*, 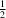 − *z*; (iv) 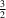 − *x*, −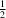 + *y*, *z*; (v) 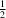 + *x*, −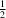 + *y*, 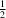 − *z*; (vi) 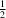 + *x*, 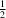 − *y*, 1 − *z*; (vii) 1 − *x*, 1 − *y*, 1 − *z*; (viii) 1 − *x*, *y*, 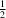 − *z*.

The title compound, [Fe(C_18_H_11_N_6_O)_2_]·2CH_3_OH, crystallizes in the ortho­rhom­bic space group *Pbcn* (No. 60) with half of the complex mol­ecule and a methanol mol­ecule in the asymmetric unit. In the complex, the two ligands meridionally bind to the central Fe^II^ ion, which lies on a twofold rotation axis through the N atoms of the heterocyclic groups, forming a pseudo-octa­hedral coordination sphere. The benzo­furan group of the ligand is rotated by 16.2 (2)° relative to the almost planar pyrazole-pyridine-triazole (pz-py-trz) fragment (r.m.s. deviation = 0.055 Å). The methanol mol­ecule forms hydrogen bonds with the trz rings (Fig. 1 ▸). The central Fe ion has a distorted octa­hedral N_6_ coordination environment formed by the nitro­gen donor atoms of the tridentate ligands. The average bond length iron–nitro­gen (<Fe—N>) of 1.957 (2) Å and the volume of the [FeN_6_] coordination polyhedron of 9.64 Å^3^ are small and afor the low-spin state of the central ion (Gütlich & Goodwin, 2004 ▸). The average trigonal distortion parameters *Σ* = Σ_1_^12^(|90 − *ϕ*_i_|), where *ϕ*_i_ is the angle N—Fe—N′, and *Θ* = Σ_1_^24^(|60 − *θ*_i_|), where *θ*_i_ is the angle generated by superposition of two opposite faces of an octa­hedron, are 90.9 and 315.2°, respectively. The calculated continuous shape measure [CShM(*O*_h_)] value relative to the ideal octa­hedral symmetry is 2.300 (Kershaw Cook *et al.*, 2015 ▸). The values indicate a pseudo-octa­hedral coordination environment [for an ideal octa­hedron *Σ* = *Θ* = CShM(*O*_h_) = 0].

## 3. Supra­molecular features

**Figure 2:**
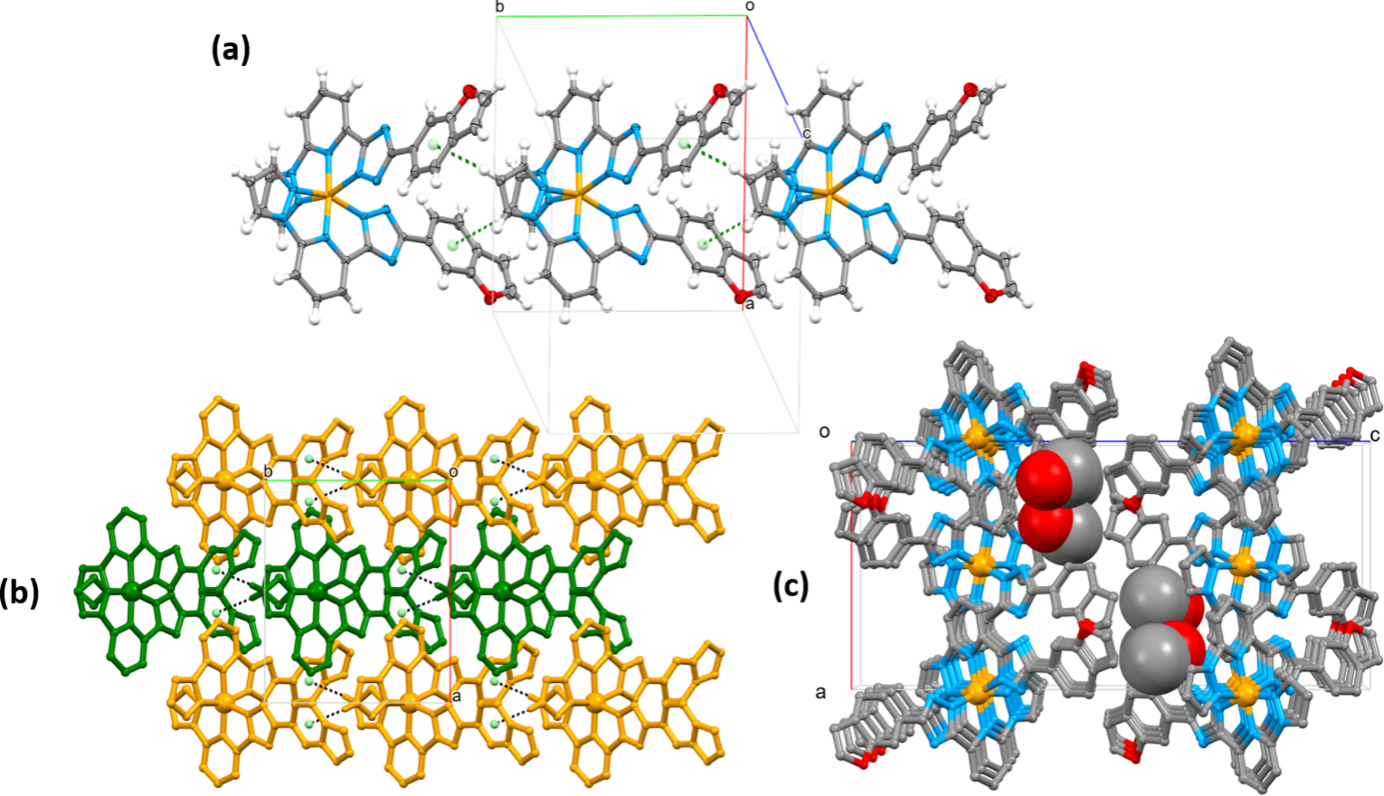
(*a*) A fragment of supra­molecular column formed by stacking of mol­ecules along the *b* axis; (*b*) Supra­molecular layers formed by stacking of the supra­molecular columns in the *ab* plane (for a better representation, each column has a different colour). Hydrogen atoms, except those in pz-groups participating in C–H⋯*C*g(π) inter­actions, are omitted for clarity; (*c*) Stacking of the layers along the *b* axis direction with the methanol mol­ecules in the voids. Hydrogen atoms are omitted for clarity.

In the crystal, the mol­ecules inter­lock by inserting the narrower end of one into the wider end of another, and inter­act through weak C—H(pz)⋯π(ph) inter­molecular contacts between the pyrazole and phenyl groups [the H2/C2⋯*C*g(ph) distance is 2.550 (2)/3.489 (2) Å]. The formed supra­molecular chains extend along the *b-*axis direction with a stacking periodicity of 10.5670 (1) Å (Fig. 2 ▸)*.* Weak inter­molecular C—H(pz, py)⋯N/C(pz, trz) inter­actions, ranging from 3.189 (2) to 3.695 (2) Å (Table 1 ▸), connect neighbouring chains into layers propagating in the *ab* plane. The voids between the layers are occupied by methanol mol­ecules, which also participate in the bonding within separate layers. The methanol mol­ecules form a strong O—H⋯N5 hydrogen bond with the deprotonated trz groups and weak C—H⋯O hydrogen bonds with the pz and py groups of the ligand. A complete list of selected inter­molecular inter­actions is provided in Table 1 ▸.

**Figure 3:**
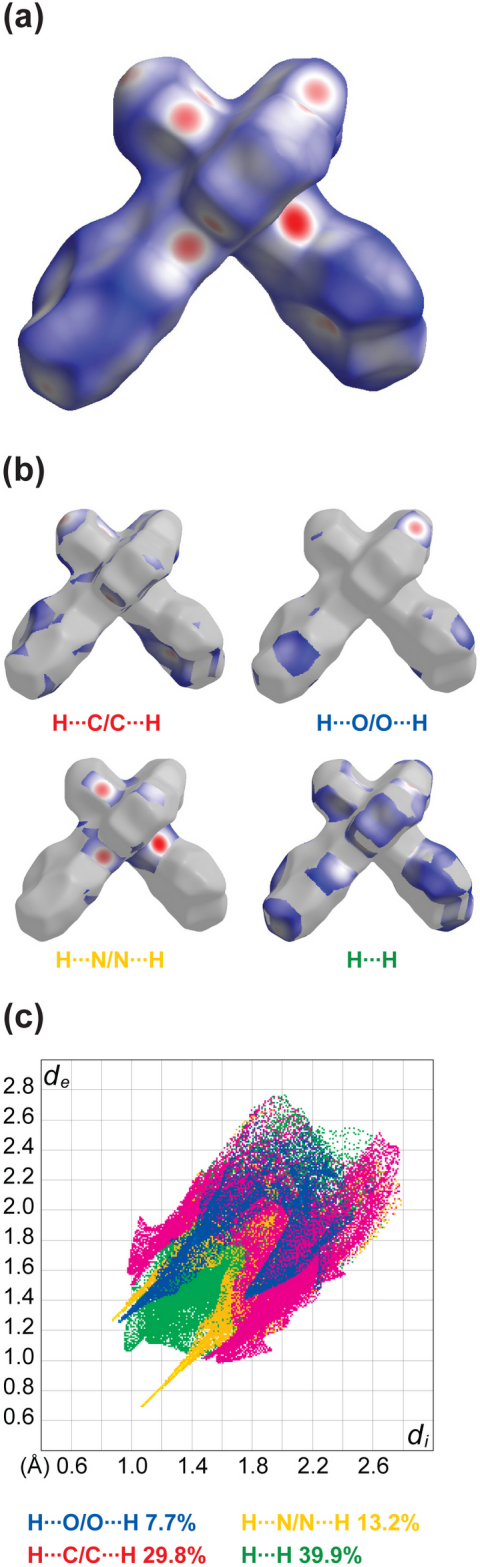
(*a*) A projection of *d*_norm_ mapped on the Hirshfeld surface identifying contact points or areas for inter­molecular inter­actions on the mol­ecule; (*b*) decomposition of the projection *d*_norm_ into the specific inter­molecular inter­actions; (*c*) decomposition of the two-dimensional fingerprint plot into specific inter­actions.

Hirshfeld surface analysis was conducted for the complex to gain a deeper understanding of the inter­actions. These inter­actions are visualized as red (*d*_norm_< vdW radii), white (*d_norm_*= vdW radii), and blue (*d*_norm_> vdW radii) spots on the *d*_norm_ surface for the compound along with fingerprint plots mapped with *d*_norm_ (where *d*_norm_*= d*_i_*+ d*_e_) and decomposed to the separate contributions (Fig. 3 ▸*a*–*c*). At 39.9%, the largest contribution to the overall crystal packing is from H⋯H inter­actions, which are located in the middle region of the fingerprint plot. H⋯C contacts contribute 29.8%, and H⋯O 7.7%, resulting in pairs of characteristic wings. The H⋯N contacts, represented by a pair of sharp spikes in the fingerprint plot, make a 13.2% contribution to the surface.

**Figure 4:**
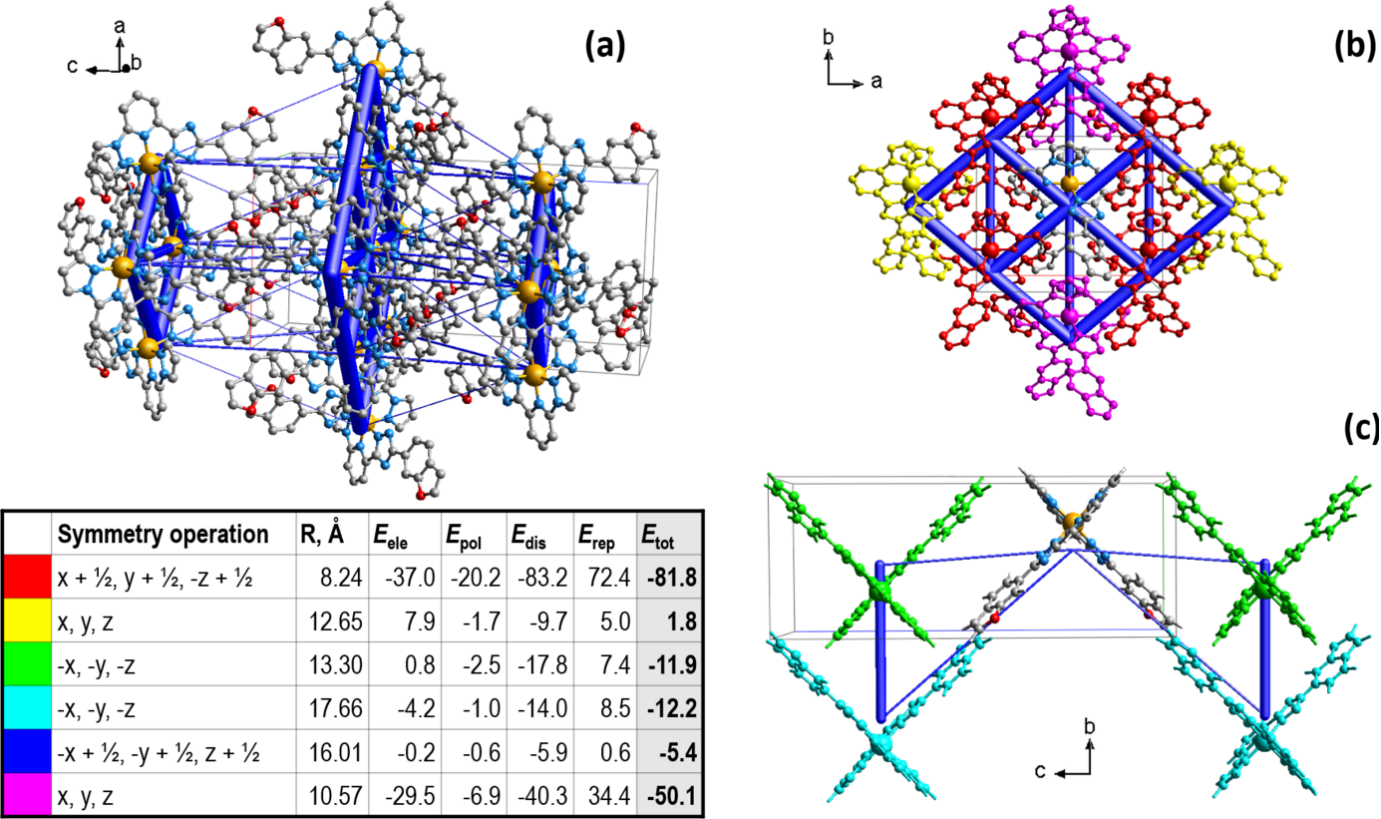
(*a*) The calculated energy frameworks, showing the total energy diagrams (*E*_tot_); (*b*) decomposition of the energy framework into the part corresponding to the intra­layer inter­actions and (*c*) inter­layer inter­actions. In the table, the corresponding colour-coded energy values *E*_tot_ are provided, including their *E*_ele_, *E*_pol_, *E*_dis_, and *E*_rep_ components. Tube size is set at 100 scale.

The energy framework (Spackman *et al.*, 2021 ▸), was calculated based on the wave function at the B3LYP/6-31G(d,p) theory level. This framework includes components such as electrostatic (*E*_ele_), polarization (*E*_pol_), repulsion (*E*_rep_), and dispersion (*E*_dis_) inter­actions. The latter dominate the contributions, underscoring their primary role for neutral mol­ecules in the crystal structure. The total energy diagram (*E*_tot_) overlaid with a fragment of the crystal structure is built using cylindrical bonds between centroids of mol­ecules, where the radii are proportional to the relative inter­action strengths (Fig. 4 ▸*a*–*c*). The overall topology of the energy framework mirrors the inter­action patterns both within and between layers, as outlined earlier. Qu­anti­tatively, the *E*_tot_ for intra­chain inter­actions is −50.1 kJ mol^−1^, while inter­chain inter­actions reach values as low as −81.8 kJ mol^−1^. Inter­layer inter­actions, in contrast, have an energy as low as −12.2 kJ mol^−1^. The figure also shows colour-coding of the inter­actions around a central reference mol­ecule, along with a table of the individual contributions to *E*_tot_.

## 4. Database survey

A search of the Cambridge Structural Database (CSD, Version 5.42, last update April 2025; Groom *et al.*, 2016 ▸) reveals several low-spin neutral Fe^II^ complexes based on asymmetric bis­azolpyridines. The selected representative compound for different pairs of azol-azol substituents are ABUFOV (Rajnák *et al.*, 2017 ▸), BEJQOA (Seredyuk *et al.*, 2022 ▸), BOWRIR (Senthil Kumar *et al.*, 2020 ▸) and XODCEB (Shiga *et al.*, 2019 ▸). Table 2 ▸ collates some key structural parameters of the complexes. Compared to the title compound, the surveyed complexes generally do not bear voluminous substituents and exhibit lower coordination sphere distortion parameters, suggesting that the bulky benzo­furan group, rigidly linked to the donor groups in the present ligand, likely induces greater deviation from an ideal octa­hedral geometry. This observation underscores the significant influence of peripheral substituents on the structural properties of such complexes.

## 5. Synthesis and crystallization

The synthesis of the title compound followed the protocol reported for a similar complex (Seredyuk *et al.*, 2022 ▸). It was produced by a layering technique in a standard test tube. The layering sequence was as follows: the bottom layer contained a solution of [Fe(*L*_2_)](BF_4_)_2_ prepared by dissolving *L* = 3-(benzo­furan-6-yl)-5-[6-(1*H*-pyrazol-1-yl)pyridin-2-yl]-4*H*-1,2,4-triazol (100 mg, 0.304 mmol) and Fe(BF_4_)_2_·6H_2_O (51 mg, 0.152 mmol) in boiling acetone, to which chloro­form (5 ml) was then added. The middle layer was a methanol–chloro­form mixture (1:10; 10 ml), which was covered by a layer of methanol (10 ml), to which 100 µl of NEt_3_ was added dropwise. The tube was sealed, and dark-red plate-like single crystals appeared after 3–4 weeks (yield *ca*. 80%). Elemental analysis calculated for C_38_H_30_FeN_12_O_4_: C, 58.92; H, 3.90; N, 21.70. Found: C, 59.14; H, 3.97; N, 22.05.

## 6. Refinement

Crystal data, data collection and structure refinement details are summarized in Table 3 ▸. The hydrogen atom H2*A* was refined freely, other H atoms were refined as riding [C—H = 0.95–0.98 Å with *U*_iso_(H) = 1.2–1.5*U*_eq_(C)].

## Supplementary Material

Crystal structure: contains datablock(s) I. DOI: 10.1107/S2056989025008655/zn2044sup1.cif

Structure factors: contains datablock(s) I. DOI: 10.1107/S2056989025008655/zn2044Isup2.hkl

Supporting information file. DOI: 10.1107/S2056989025008655/zn2044Isup3.cdx

CCDC reference: 2492851

Additional supporting information:  crystallographic information; 3D view; checkCIF report

## Figures and Tables

**Table 1:** Hydrogen-bond and short contact geometry (Å, °)

*D*—H⋯*A*	*D*—H	H⋯*A*	*D*⋯*A*	*D*—H⋯*A*
O2—H2*A*⋯N5	0.85 (3)	1.89 (2)	2.750 (2)	178 (3)
C1—H1⋯N6^i^	0.95	2.27	3.189 (2)	163
C3—H3⋯O2^ii^	0.95	2.29	3.208 (2)	161
C5—H5⋯O2^ii^	0.95	2.46	3.386 (2)	164
C5—H5⋯N5^ii^	0.95	1.90	3.463 (2)	129
C7—H7⋯C1^iii^	0.95	2.63	3.537 (2)	159
C19—H19*A*⋯N2^iv^	0.97	2.74	3.491 (2)	133
C19—H19*A*⋯C3^iv^	0.97	2.89	3.695 (2)	140
C6⋯C3^v^	–	–	3.482 (2)	–
C7⋯C3^v^	–	–	3.498 (2)	–

Symmetry codes: (i) 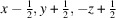; (ii) 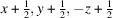; (iii) 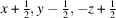; (iv) 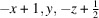; (v) 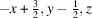.

**Table 2:** Computed distortion indices (Å, °) for the title compound and for similar complexes reported in the literature.

CSD Refcode	azol 1/azol 2	<Fe—N>	*Σ*	*Θ*	CShM(*O*_h_)
Title compound	1,2,4-triazole/pyrazole	1.957	90.9	315.2	2.300
BEJQOA	1,2,4-triazole/pyrazole	1.946	87.5	308.9	2.163
ABUFOV	benzimidazole/benzimidazole	1.937	80.1	262.7	1.753
BOWRIR	tetra­zole/pyrazole	1.934	89.7	287.4	2.043
XODCEB	benzimidazole/pyrazole	1.950	87.5	276.6	1.925

**Table 3:** Experimental details

Crystal data	
Chemical formula	[Fe(C_18_H_11_N_6_O)_2_]·2CH_4_O
*M*_r_	774.59
Crystal system, space group	Orthorhombic, *Pbcn*
Temperature (K)	100
*a*, *b*, *c* (Å)	12.64747 (16), 10.56703 (12), 26.4991 (4)
*V* (Å^3^)	3541.51 (8)
*Z*	4
Radiation type	Cu *K*α
μ (mm^−1^)	3.92
Crystal size (mm)	0.2 × 0.10 × 0.02

Data collection	
Diffractometer	Rigaku R-AXIS Spider
Absorption correction	Multi-scan (*CrysAlis PRO*; Rigaku OD, 2024 ▸)
*T*_min_, *T*_max_	0.534, 1.000
No. of measured, independent and observed [*I* > 2σ(*I*)] reflections	14149, 3464, 3112
*R*_int_	0.035
(sin θ/λ)_max_ (Å^−1^)	0.632

Refinement	
*R*[*F*^2^ > 2σ(*F*^2^)], *wR*(*F*^2^), *S*	0.037, 0.097, 1.05
No. of reflections	3464
No. of parameters	267
H-atom treatment	H atoms treated by a mixture of independent and constrained refinement
Δρ_max_, Δρ_min_ (e Å^−3^)	0.38, −0.30

Computer programs: *CrysAlis PRO* (Rigaku OD, 2024 ▸), *OLEX2.solve* 1.5 (Bourhis *et al.*, 2015 ▸), *SHELXL2018/3* (Sheldrick, 2015 ▸) and *OLEX2* (Dolomanov *et al.*, 2009 ▸).

## supplementary crystallographic information

### Bis{3-(benzofuran-6-yl)-5-[6-(1H-pyrazol-1-yl)pyridin-2-yl]-1H-1,2,4-triazol-1-ido}iron(II) methanol disolvate Crystal data

**Table:**

[Fe(C_18_H_11_N_6_O)_2_]·2CH_4_O	*D*_x_ = 1.453 Mg m^−^^3^
*M**_r_* = 774.59	Cu *K*α radiation, λ = 1.54184 Å
Orthorhombic, *P**b**c**n*	Cell parameters from 1992 reflections
*a* = 12.64747 (16) Å	θ = 2.6–21.9°
*b* = 10.56703 (12) Å	µ = 3.92 mm^−^^1^
*c* = 26.4991 (4) Å	*T* = 100 K
*V* = 3541.51 (8) Å^3^	Plate, clear dark red
*Z* = 4	0.2 × 0.1 × 0.02 mm
*F*(000) = 1600	

### Bis{3-(benzofuran-6-yl)-5-[6-(1H-pyrazol-1-yl)pyridin-2-yl]-1H-1,2,4-triazol-1-ido}iron(II) methanol disolvate Data collection

**Table:**

Rigaku R-AXIS Spider diffractometer	3464 independent reflections
Radiation source: sealed X-ray tube	3112 reflections with *I* > 2σ(*I*)
Graphite monochromator	*R*_int_ = 0.035
ω scans	θ_max_ = 77.0°, θ_min_ = 3.3°
Absorption correction: multi-scan (CrysAlisPro; Rigaku OD, 2024)	*h* = −14→15
*T*_min_ = 0.534, *T*_max_ = 1.000	*k* = −10→12
14149 measured reflections	*l* = −32→31

### Bis{3-(benzofuran-6-yl)-5-[6-(1H-pyrazol-1-yl)pyridin-2-yl]-1H-1,2,4-triazol-1-ido}iron(II) methanol disolvate Refinement

**Table:**

Refinement on *F*^2^	Primary atom site location: iterative
Least-squares matrix: full	Hydrogen site location: mixed
*R*[*F*^2^ > 2σ(*F*^2^)] = 0.037	H atoms treated by a mixture of independent and constrained refinement
*wR*(*F*^2^) = 0.097	*w* = 1/[σ^2^(*F*_o_^2^) + (0.0512*P*)^2^ + 1.8481*P*] where *P* = (*F*_o_^2^ + 2*F*_c_^2^)/3
*S* = 1.05	(Δ/σ)_max_ = 0.001
3464 reflections	Δρ_max_ = 0.38 e Å^−^^3^
267 parameters	Δρ_min_ = −0.30 e Å^−^^3^
0 restraints	

### Bis{3-(benzofuran-6-yl)-5-[6-(1H-pyrazol-1-yl)pyridin-2-yl]-1H-1,2,4-triazol-1-ido}iron(II) methanol disolvate Special details

**Table:**

Geometry. All esds (except the esd in the dihedral angle between two l.s. planes) are estimated using the full covariance matrix. The cell esds are taken into account individually in the estimation of esds in distances, angles and torsion angles; correlations between esds in cell parameters are only used when they are defined by crystal symmetry. An approximate (isotropic) treatment of cell esds is used for estimating esds involving l.s. planes.

### Bis{3-(benzofuran-6-yl)-5-[6-(1H-pyrazol-1-yl)pyridin-2-yl]-1H-1,2,4-triazol-1-ido}iron(II) methanol disolvate Fractional atomic coordinates and isotropic or equivalent isotropic displacement parameters (Å^2^)

**Table:**

	*x*	*y*	*z*	*U*_iso_*/*U*_eq_	
Fe1	0.500000	0.71753 (3)	0.250000	0.01699 (12)	
N2	0.60321 (11)	0.88309 (13)	0.18316 (5)	0.0211 (3)	
N5	0.51474 (11)	0.50739 (14)	0.33332 (6)	0.0212 (3)	
N1	0.50229 (10)	0.84881 (14)	0.19681 (6)	0.0201 (3)	
N6	0.69158 (11)	0.47812 (14)	0.32708 (6)	0.0248 (3)	
O2	0.33165 (11)	0.58628 (13)	0.37969 (6)	0.0337 (3)	
H2A	0.388 (2)	0.560 (3)	0.3652 (11)	0.051*	
C10	0.60031 (14)	0.44450 (16)	0.34999 (7)	0.0234 (4)	
N4	0.55298 (11)	0.58772 (13)	0.29740 (5)	0.0200 (3)	
O1	0.76510 (15)	0.11184 (16)	0.45227 (8)	0.0592 (5)	
N3	0.64849 (12)	0.72535 (13)	0.23605 (6)	0.0192 (3)	
C3	0.60208 (15)	0.97836 (16)	0.14857 (7)	0.0252 (4)	
H3	0.661738	1.018159	0.133646	0.028 (5)*	
C1	0.43939 (14)	0.92266 (16)	0.16998 (7)	0.0231 (4)	
H1	0.364343	0.920088	0.171141	0.018 (5)*	
C9	0.65775 (14)	0.56733 (16)	0.29510 (7)	0.0219 (4)	
C14	0.59610 (19)	0.17309 (18)	0.46954 (7)	0.0370 (5)	
C15	0.50599 (18)	0.2460 (2)	0.46065 (8)	0.0368 (5)	
H15	0.444813	0.236306	0.481086	0.034 (6)*	
C11	0.59762 (15)	0.34926 (16)	0.39076 (7)	0.0255 (4)	
C4	0.68561 (14)	0.81295 (16)	0.20461 (6)	0.0209 (3)	
C8	0.71692 (14)	0.64890 (16)	0.26088 (7)	0.0216 (4)	
C2	0.49829 (14)	1.00533 (19)	0.13958 (7)	0.0264 (4)	
H2	0.471293	1.067668	0.117193	0.035 (6)*	
C13	0.68376 (18)	0.19108 (19)	0.43879 (8)	0.0375 (5)	
C5	0.79190 (14)	0.83074 (17)	0.19502 (7)	0.0249 (4)	
H5	0.816012	0.894204	0.172386	0.035 (6)*	
C6	0.86160 (14)	0.75147 (18)	0.22005 (8)	0.0279 (4)	
H6	0.935455	0.759817	0.214414	0.028 (5)*	
C12	0.68793 (17)	0.27699 (18)	0.39928 (9)	0.0349 (5)	
H12	0.749420	0.286094	0.379018	0.035 (6)*	
C19	0.3624 (2)	0.6729 (2)	0.41714 (9)	0.0448 (6)	
H19A	0.408898	0.737042	0.402292	0.068 (9)*	
H19B	0.400247	0.628001	0.443998	0.064 (9)*	
H19C	0.299544	0.713942	0.431245	0.071 (10)*	
C7	0.82468 (16)	0.65977 (19)	0.25333 (7)	0.0273 (4)	
H7	0.872601	0.605658	0.270552	0.029 (5)*	
C16	0.50739 (16)	0.33329 (19)	0.42130 (8)	0.0303 (4)	
H16	0.446288	0.383066	0.414909	0.031 (6)*	
C18	0.6261 (3)	0.0745 (2)	0.50447 (9)	0.0503 (7)	
H18	0.583948	0.039318	0.530606	0.066 (9)*	
C17	0.7255 (3)	0.0429 (2)	0.49248 (11)	0.0611 (8)	
H17	0.764801	−0.020237	0.509770	0.073 (10)*	

### Bis{3-(benzofuran-6-yl)-5-[6-(1H-pyrazol-1-yl)pyridin-2-yl]-1H-1,2,4-triazol-1-ido}iron(II) methanol disolvate Atomic displacement parameters (Å^2^)

**Table:**

	*U*^11^	*U*^22^	*U*^33^	*U*^12^	*U*^13^	*U*^23^
Fe1	0.0157 (2)	0.0172 (2)	0.0181 (2)	0.000	−0.00027 (13)	0.000
N2	0.0203 (7)	0.0218 (7)	0.0213 (7)	0.0000 (6)	0.0010 (6)	0.0024 (6)
N5	0.0224 (7)	0.0200 (7)	0.0212 (7)	−0.0031 (6)	0.0002 (6)	0.0033 (6)
N1	0.0184 (7)	0.0204 (7)	0.0214 (7)	0.0004 (5)	0.0001 (5)	−0.0016 (6)
N6	0.0224 (7)	0.0235 (7)	0.0286 (8)	0.0000 (6)	−0.0013 (6)	0.0059 (6)
O2	0.0288 (7)	0.0369 (7)	0.0354 (8)	−0.0025 (6)	0.0023 (6)	−0.0099 (6)
C10	0.0232 (9)	0.0212 (8)	0.0258 (9)	−0.0024 (7)	−0.0018 (7)	0.0017 (7)
N4	0.0183 (7)	0.0194 (7)	0.0222 (7)	−0.0020 (5)	−0.0005 (5)	0.0014 (6)
O1	0.0574 (11)	0.0473 (9)	0.0729 (12)	0.0010 (8)	−0.0213 (9)	0.0316 (9)
N3	0.0193 (7)	0.0188 (7)	0.0195 (7)	−0.0009 (6)	−0.0001 (6)	0.0003 (5)
C3	0.0303 (9)	0.0224 (8)	0.0229 (9)	0.0005 (7)	0.0014 (7)	0.0047 (7)
C1	0.0230 (9)	0.0233 (8)	0.0230 (8)	0.0041 (7)	−0.0019 (7)	−0.0016 (7)
C9	0.0211 (8)	0.0211 (8)	0.0234 (8)	0.0002 (7)	−0.0014 (7)	0.0028 (7)
C14	0.0639 (15)	0.0252 (9)	0.0218 (9)	−0.0096 (10)	−0.0079 (9)	0.0020 (7)
C15	0.0570 (15)	0.0290 (10)	0.0243 (10)	−0.0052 (9)	0.0058 (9)	0.0013 (8)
C11	0.0314 (10)	0.0217 (8)	0.0234 (9)	−0.0050 (7)	−0.0053 (7)	0.0025 (7)
C4	0.0227 (9)	0.0207 (8)	0.0193 (8)	0.0009 (7)	−0.0007 (7)	0.0009 (6)
C8	0.0210 (8)	0.0209 (8)	0.0230 (8)	0.0014 (7)	−0.0014 (7)	0.0011 (7)
C2	0.0309 (10)	0.0257 (9)	0.0228 (9)	0.0052 (7)	−0.0017 (7)	0.0028 (7)
C13	0.0442 (12)	0.0278 (9)	0.0405 (12)	−0.0042 (9)	−0.0168 (10)	0.0085 (9)
C5	0.0234 (9)	0.0254 (9)	0.0261 (9)	−0.0028 (7)	0.0020 (7)	0.0049 (7)
C6	0.0177 (9)	0.0313 (9)	0.0347 (10)	−0.0005 (8)	0.0014 (7)	0.0048 (8)
C12	0.0333 (11)	0.0309 (10)	0.0405 (12)	−0.0056 (8)	−0.0070 (9)	0.0119 (9)
C19	0.0680 (16)	0.0340 (11)	0.0325 (11)	−0.0132 (11)	0.0105 (11)	−0.0067 (9)
C7	0.0219 (9)	0.0280 (9)	0.0320 (10)	0.0019 (8)	−0.0016 (7)	0.0064 (8)
C16	0.0409 (11)	0.0245 (9)	0.0254 (10)	−0.0021 (8)	0.0026 (8)	0.0021 (8)
C18	0.088 (2)	0.0333 (12)	0.0300 (11)	−0.0088 (13)	−0.0149 (12)	0.0091 (9)
C17	0.084 (2)	0.0421 (13)	0.0570 (16)	−0.0069 (14)	−0.0303 (15)	0.0262 (12)

### Bis{3-(benzofuran-6-yl)-5-[6-(1H-pyrazol-1-yl)pyridin-2-yl]-1H-1,2,4-triazol-1-ido}iron(II) methanol disolvate Geometric parameters (Å, º)

**Table:**

Fe1—N1^i^	1.9778 (15)	C9—C8	1.458 (2)
Fe1—N1	1.9778 (15)	C14—C15	1.396 (3)
Fe1—N4	1.9770 (14)	C14—C13	1.389 (3)
Fe1—N4^i^	1.9770 (14)	C14—C18	1.444 (3)
Fe1—N3^i^	1.9158 (15)	C15—H15	0.9500
Fe1—N3	1.9158 (15)	C15—C16	1.392 (3)
N2—N1	1.375 (2)	C11—C12	1.392 (3)
N2—C3	1.362 (2)	C11—C16	1.409 (3)
N2—C4	1.399 (2)	C4—C5	1.381 (2)
N5—C10	1.345 (2)	C8—C7	1.382 (3)
N5—N4	1.364 (2)	C2—H2	0.9500
N1—C1	1.322 (2)	C13—C12	1.387 (3)
N6—C10	1.352 (2)	C5—H5	0.9500
N6—C9	1.338 (2)	C5—C6	1.385 (3)
O2—H2A	0.85 (3)	C6—H6	0.9500
O2—C19	1.405 (3)	C6—C7	1.391 (3)
C10—C11	1.477 (2)	C12—H12	0.9500
N4—C9	1.344 (2)	C19—H19A	0.9800
O1—C13	1.374 (3)	C19—H19B	0.9800
O1—C17	1.384 (3)	C19—H19C	0.9800
N3—C4	1.331 (2)	C7—H7	0.9500
N3—C8	1.354 (2)	C16—H16	0.9500
C3—H3	0.9500	C18—H18	0.9500
C3—C2	1.364 (3)	C18—C17	1.339 (4)
C1—H1	0.9500	C17—H17	0.9500
C1—C2	1.403 (3)		
			
N1^i^—Fe1—N1	90.92 (9)	C14—C15—H15	120.6
N4^i^—Fe1—N1	92.23 (6)	C16—C15—C14	118.8 (2)
N4—Fe1—N1^i^	92.23 (6)	C16—C15—H15	120.6
N4—Fe1—N1	159.14 (6)	C12—C11—C10	118.26 (17)
N4^i^—Fe1—N1^i^	159.14 (5)	C12—C11—C16	120.36 (18)
N4—Fe1—N4^i^	92.13 (8)	C16—C11—C10	121.36 (17)
N3^i^—Fe1—N1^i^	79.51 (6)	N3—C4—N2	111.09 (15)
N3—Fe1—N1	79.51 (6)	N3—C4—C5	123.59 (16)
N3—Fe1—N1^i^	96.99 (6)	C5—C4—N2	125.31 (16)
N3^i^—Fe1—N1	96.98 (6)	N3—C8—C9	109.07 (15)
N3^i^—Fe1—N4^i^	79.64 (6)	N3—C8—C7	120.68 (16)
N3^i^—Fe1—N4	103.86 (6)	C7—C8—C9	130.18 (17)
N3—Fe1—N4	79.64 (6)	C3—C2—C1	106.30 (16)
N3—Fe1—N4^i^	103.86 (6)	C3—C2—H2	126.8
N3—Fe1—N3^i^	175.06 (8)	C1—C2—H2	126.8
N1—N2—C4	116.41 (14)	O1—C13—C14	111.21 (19)
C3—N2—N1	111.23 (14)	O1—C13—C12	124.5 (2)
C3—N2—C4	132.29 (15)	C12—C13—C14	124.3 (2)
C10—N5—N4	104.56 (13)	C4—C5—H5	121.7
N2—N1—Fe1	112.69 (10)	C4—C5—C6	116.69 (16)
C1—N1—Fe1	142.05 (12)	C6—C5—H5	121.7
C1—N1—N2	105.17 (14)	C5—C6—H6	119.6
C9—N6—C10	101.33 (14)	C5—C6—C7	120.74 (17)
C19—O2—H2A	107.2 (19)	C7—C6—H6	119.6
N5—C10—N6	114.20 (15)	C11—C12—H12	121.6
N5—C10—C11	123.95 (16)	C13—C12—C11	116.8 (2)
N6—C10—C11	121.82 (16)	C13—C12—H12	121.6
N5—N4—Fe1	139.02 (11)	O2—C19—H19A	109.5
C9—N4—Fe1	114.63 (11)	O2—C19—H19B	109.5
C9—N4—N5	106.35 (13)	O2—C19—H19C	109.5
C13—O1—C17	104.5 (2)	H19A—C19—H19B	109.5
C4—N3—Fe1	119.77 (12)	H19A—C19—H19C	109.5
C4—N3—C8	119.57 (15)	H19B—C19—H19C	109.5
C8—N3—Fe1	120.46 (12)	C8—C7—C6	118.72 (17)
N2—C3—H3	126.8	C8—C7—H7	120.6
N2—C3—C2	106.38 (16)	C6—C7—H7	120.6
C2—C3—H3	126.8	C15—C16—C11	121.32 (19)
N1—C1—H1	124.5	C15—C16—H16	119.3
N1—C1—C2	110.91 (16)	C11—C16—H16	119.3
C2—C1—H1	124.5	C14—C18—H18	127.0
N6—C9—N4	113.56 (15)	C17—C18—C14	105.9 (2)
N6—C9—C8	130.27 (16)	C17—C18—H18	127.0
N4—C9—C8	116.07 (15)	O1—C17—H17	123.5
C15—C14—C18	136.1 (2)	C18—C17—O1	113.0 (2)
C13—C14—C15	118.49 (18)	C18—C17—H17	123.5
C13—C14—C18	105.4 (2)		
			
Fe1—N1—C1—C2	175.25 (15)	N3—C8—C7—C6	0.5 (3)
Fe1—N4—C9—N6	−179.26 (12)	C3—N2—N1—Fe1	−176.63 (11)
Fe1—N4—C9—C8	3.9 (2)	C3—N2—N1—C1	0.66 (19)
Fe1—N3—C4—N2	−4.37 (19)	C3—N2—C4—N3	−177.97 (17)
Fe1—N3—C4—C5	175.56 (14)	C3—N2—C4—C5	2.1 (3)
Fe1—N3—C8—C9	1.54 (19)	C9—N6—C10—N5	0.5 (2)
Fe1—N3—C8—C7	−175.82 (14)	C9—N6—C10—C11	−177.82 (16)
N2—N1—C1—C2	−0.70 (19)	C9—C8—C7—C6	−176.21 (18)
N2—C3—C2—C1	−0.1 (2)	C14—C15—C16—C11	−0.3 (3)
N2—C4—C5—C6	179.99 (17)	C14—C13—C12—C11	0.3 (3)
N5—C10—C11—C12	170.28 (18)	C14—C18—C17—O1	−0.1 (3)
N5—C10—C11—C16	−11.3 (3)	C15—C14—C13—O1	−179.64 (19)
N5—N4—C9—N6	0.3 (2)	C15—C14—C13—C12	−0.3 (3)
N5—N4—C9—C8	−176.53 (14)	C15—C14—C18—C17	179.4 (3)
N1—N2—C3—C2	−0.4 (2)	C4—N2—N1—Fe1	6.09 (18)
N1—N2—C4—N3	−1.4 (2)	C4—N2—N1—C1	−176.61 (14)
N1—N2—C4—C5	178.66 (17)	C4—N2—C3—C2	176.33 (18)
N1—C1—C2—C3	0.5 (2)	C4—N3—C8—C9	176.42 (15)
N6—C10—C11—C12	−11.6 (3)	C4—N3—C8—C7	−0.9 (3)
N6—C10—C11—C16	166.86 (18)	C4—C5—C6—C7	−0.5 (3)
N6—C9—C8—N3	−179.67 (17)	C8—N3—C4—N2	−179.28 (14)
N6—C9—C8—C7	−2.6 (3)	C8—N3—C4—C5	0.6 (3)
C10—N5—N4—Fe1	179.40 (14)	C13—O1—C17—C18	−0.1 (3)
C10—N5—N4—C9	0.06 (18)	C13—C14—C15—C16	0.3 (3)
C10—N6—C9—N4	−0.5 (2)	C13—C14—C18—C17	0.2 (3)
C10—N6—C9—C8	175.77 (18)	C5—C6—C7—C8	0.2 (3)
C10—C11—C12—C13	178.16 (17)	C12—C11—C16—C15	0.3 (3)
C10—C11—C16—C15	−178.12 (18)	C16—C11—C12—C13	−0.3 (3)
N4—N5—C10—N6	−0.4 (2)	C18—C14—C15—C16	−178.8 (2)
N4—N5—C10—C11	177.92 (16)	C18—C14—C13—O1	−0.3 (2)
N4—C9—C8—N3	−3.5 (2)	C18—C14—C13—C12	179.1 (2)
N4—C9—C8—C7	173.51 (19)	C17—O1—C13—C14	0.2 (3)
O1—C13—C12—C11	179.6 (2)	C17—O1—C13—C12	−179.1 (2)
N3—C4—C5—C6	0.1 (3)		

Symmetry code: (i) −*x*+1, *y*, −*z*+1/2.

### Bis{3-(benzofuran-6-yl)-5-[6-(1H-pyrazol-1-yl)pyridin-2-yl]-1H-1,2,4-triazol-1-ido}iron(II) methanol disolvate Hydrogen-bond geometry (Å, º)

**Table:**

*D*—H···*A*	*D*—H	H···*A*	*D*···*A*	*D*—H···*A*
O2—H2*A*···N5	0.85 (3)	1.89 (2)	2.750 (2)	178 (3)
C1—H1···N6^ii^	0.95	2.27	3.189 (2)	163
C3—H3···O2^iii^	0.95	2.29	3.208 (2)	161
C5—H5···O2^iii^	0.95	2.46	3.386 (2)	164
C5—H5···N5^iii^	0.95	1.90	3.463 (2)	129
C7—H7···C1^iv^	0.95	2.63	3.537 (2)	159
C19—H19*A*···N2^i^	0.97	2.74	3.491 (2)	133
C19—H19*A*···C3^i^	0.97	2.89	3.695 (2)	140
C6···C3^v^	–	–	3.482 (2)	–
C7···C3^v^	–	–	3.498 (2)	–

Symmetry codes: (i) −*x*+1, *y*, −*z*+1/2; (ii) *x*−1/2, *y*+1/2, −*z*+1/2; (iii) *x*+1/2, *y*+1/2, −*z*+1/2; (iv) *x*+1/2, *y*−1/2, −*z*+1/2; (v) −*x*+3/2, *y*−1/2, *z*.
